# Strengthening the genomic surveillance of *Francisella tularensis* by using culture-free whole-genome sequencing from biological samples

**DOI:** 10.3389/fmicb.2023.1277468

**Published:** 2024-01-05

**Authors:** Joana Isidro, Raquel Escudero, Juan José Luque-Larena, Miguel Pinto, Vítor Borges, Rosa González-Martín-Niño, Sílvia Duarte, Luís Vieira, François Mougeot, Dolors Vidal, Daniel Herrera-Rodríguez, Ruth Rodríguez-Pastor, Silvia Herrero-Cófreces, Fernando Jubete-Tazo, João Paulo Gomes, Isabel Lopes de Carvalho

**Affiliations:** ^1^Genomics and Bioinformatics Unit, National Institute of Health Doutor Ricardo Jorge (INSA), Lisbon, Portugal; ^2^Reference and Research Laboratory on Special Pathogens, National Centre for Microbiology (CNM), Carlos II Health Institute (ISCIII), Madrid, Spain; ^3^Departamento de Ciencias Agroforestales, Instituto Universitario de Investigación en Gestión Forestal Sostenible (iuFOR), E.T.S. Ingenierías Agrarias, Universidad de Valladolid, Palencia, Spain; ^4^Technology and Innovation Unit, Department of Human Genetics, National Institute of Health Doutor Ricardo Jorge (INSA), Lisbon, Portugal; ^5^Instituto de Investigación en Recursos Cinegéticos (IREC-CSIC, UCLM, JCCM), Ciudad Real, Spain; ^6^Área de Microbiología, Facultad de Medicina, Universidad de Catilla-La Mancha (UCLM), Ciudad Real, Spain; ^7^Department of Parasitology, Veterinary Faculty, University of Zaragoza, Zaragoza, Spain, Zaragoza, Spain; ^8^Departamento de Parasitología, Facultad de Veterinaria, Universidad de Zaragoza, Instituto Agroalimentario de Aragón-IA2 (Universidad de Zaragoza-CITA), Zaragoza, Spain; ^9^Veterinary and Animal Research Center (CECAV), Faculty of Veterinary Medicine, Lusófona University, Lisbon, Portugal; ^10^Emergency and Biopreparedness Unit, National Institute of Health Doutor Ricardo Jorge, Lisbon, Portugal

**Keywords:** *Francisella tularensis*, WGS, RNA oligonucleotide baits, *Microtus arvalis*, SureSelect

## Abstract

**Introduction:**

*Francisella tularensis* is a highly infectious bacterium that causes the zoonotic disease tularemia. The development of genotyping methods, especially those based on whole-genome sequencing (WGS), has recently increased the knowledge on the epidemiology of this disease. However, due to the difficulties associated with the growth and isolation of this fastidious pathogen in culture, the availability of strains and subsequently WGS data is still limited.

**Methods:**

To surpass these constraints, we aimed to implement a culture-free approach to capture and sequence *F. tularensis* genomes directly from complex samples. Biological samples obtained from 50 common voles and 13 Iberian hares collected in Spain were confirmed as positive for *F. tularensis subsp. holarctica* and subjected to a WGS target capture and enrichment protocol, using RNA oligonucleotide baits designed to cover *F. tularensis* genomic diversity.

**Results:**

We obtained full genome sequences of *F. tularensis* from 13 animals (20.6%), two of which had mixed infections with distinct genotypes, and achieved a higher success rate when compared with culture-dependent WGS (only successful for two animals). The new genomes belonged to different clades commonly identified in Europe (B.49, B.51 and B.262) and subclades. Despite being phylogenetically closely related to other genomes from Spain, the detected clusters were often found in other countries. A comprehensive phylogenetic analysis, integrating 599 *F. tularensis* subsp. holarctica genomes, showed that most (sub)clades are found in both humans and animals and that closely related strains are found in different, and often geographically distant, countries.

**Discussion:**

Overall, we show that the implemented culture-free WGS methodology yields timely, complete and high-quality genomic data of *F. tularensis*, being a highly valuable approach to promote and potentiate the genomic surveillance of *F. tularensis* and ultimately increase the knowledge on the genomics, ecology and epidemiology of this highly infectious pathogen.

## 1 Introduction

*Francisella tularensis* is a facultative intracellular bacterium causing the zoonotic disease tularemia. It is considered a potential bioterrorist agent due to its very low infective dose and considerable stability in aerosols, being classified as a Category A biological agent by Centers for Disease Control and Prevention (CDC) (n.d.). *F. tularensis* may be transmitted to humans by a number of different routes, including handling infected animals, ingestion of contaminated food or water, inhalation of infective aerosols or arthropod bites (ticks and insects) (Petersen et al., 2009). In nature, *F. tularensis* has been detected in many wild species including lagomorphs, rodents, insectivores, carnivores, ungulates, marsupials, birds, amphibians, fish, and invertebrates. In Northwest (NW) Spain, the region of Castilla-y-León is an endemic hotspot of the disease in southern Europe, accumulating more than 1,000 human cases since 1997 and its epidemiology is mainly associated with population outbreaks of common voles, *Microtus arvalis*, in intensive farming landscapes (Luque-Larena et al., 2017; Herrero-Cófreces et al., 2021).

There are two clinically relevant subspecies of *F. tularensis*: *F. tularensis* subsp. *tularensis* and *F. tularensis* subsp. *holarctica*. Only the less pathogenic subspecies *holarctica* has been detected in Europe (Dennis et al., 2001; Carvalho et al., 2014). The diagnosis of tularemia is complex due both to the nonspecific nature of the initial symptoms and to the fact that *F. tularensis* is difficult to culture (Doern, 2000), showing slow growth rates, with individual colonies on nonselective optimized agar plates usually requiring two to four days of incubation to be visible (Aloni-Grinstein et al., 2017).

Environmental studies on the distribution of *Francisella* spp. are hampered by the frequency of other species of *Francisella* such as *Francisella*-like endosymbionts and other so far not proven pathogenic species that can produce a misleading positive result (Escudero et al., 2008). Efforts have been made to improve methods for the discrimination on *Francisella* species.

Considering the clonal nature of this species, only molecular methods with high discriminatory power, such as whole genome sequencing (WGS), allow to distinguish between closely related subpopulations at strain level (Johansson and Petersen, 2010; Dwibedi et al., 2016). The genotyping of *F. tularensis* strains is mostly based on the identification of canonical SNPs defined on a whole-genome level (Kevin et al., 2020). However, despite rapidly accumulating knowledge, the phylogeography of the pathogen is still poorly understood due to the low availability of isolates for WGS and consequent lack of *F. tularensis* genomic data in many tularemia-endemic countries (Shevtsov et al., 2021).

In this study, we implemented a culture-free approach for capturing and sequencing *F. tularensis* genomes directly from complex biological samples, passing the constraints associated with the isolation and culture of this fastidious pathogen. Ultimately, we aimed to increase the knowledge on *F. tularensis* genomic epidemiology not only in Spain but also on a global scale by an integrative analysis with genomes from multiple countries.

## 2 Methods

### 2.1 Sample collection and selection

In NW Spain, the common vole is a key host and spillover agent of tularemia (Herrero-Cófreces et al., 2021). Vole samples were obtained from long-term ecological studies in which animals are captured in the field (Rodríguez-Pastor et al., 2017). Voles were live-trapped using Sherman© traps (8 cm × 9 cm × 23 cm, LFAHD Sherman©) baited with carrots and apples, which were set open in the morning and retrieved 24 h later. Captures were performed every four months (March, July and November) between July 2009 and July 2015. Captured voles were taken alive to the laboratory and euthanized using a CO_2_ cabinet, following a protocol approved by the ethics committee from the University of Valladolid (authorization code: 4801646). Iberian hare samples were obtained from hunters and collected in the field during two hunting seasons (from October 2016 to February 2018). Subsequently, necropsies were performed in a microbiological safety cabinet, collecting different samples in aseptic conditions (mainly liver and spleen) for the detection of *F. tularensis*. All animals were captured from the provinces of Palencia (42°01′N, 4°42′W), Valladolid (41°34′N, 5°14′W) and Zamora (41°50′N, 5°36′W).

In the present study, the selected biological samples (liver, spleen and lung) were obtained from 50 *M. arvalis* that had tested positive for *F. tularensis* by real time-PCR, mostly with low Ct results. Moreover, DNAs extracted from homogenates of spleen and liver from 13 Iberian hares were tested as well, with signs suggestive of tularemia (i.e., hepatomegaly, splenomegaly or foci of necrosis in the selected tissues).

### 2.2 Culture of the samples

To culture the etiological agent, samples from 50 voles, consisting of lung, liver and spleen of each animal, were inoculated separately in culture media. Each tissue was punctured 10 to 20 times with a sterile wooden stick. Tissue adhering to the stick was transferred to chocolate agar PolyViteX (Biomerieux) and then a 1-μL sterile loop was used to streak the plate for colony isolation. All plates were incubated at 37°C for 5 more days and checked daily for characteristic *F. tularensis* growth (Petersen et al., 2004).

### 2.3 DNA extraction and real-time PCR

For cultures, DNA was extracted and purified using QIAGEN^®^ Genomic-tip 100/G columns and QIAGEN^®^ Genomic DNA Buffer sets from suspensions calibrated to 3 McFarland units according to the manufacturer’s recommendations. For the biological samples, DNA from a homogenized mixture of liver and spleen (≈25 mg) was extracted by standard procedures using the QIAamp DNA Mini Kit (QIAGEN, Valencia, CA, United States).

Samples were tested using a real-time multitarget TaqMan PCR, using *tul4* and *ISFtu2* assays, and genome equivalents (GEs) concentration was inferred as previously described (Versage et al., 2003). Positive samples were further tested using real-time TaqMan PCR assays which differentiate between *F. tularensis* subsp. *tularensis* (type A) and *F. tularensis* subsp. *holarctica* (type B) (Kugeler et al., 2006). Moreover, a phylogenetically informative region of lipoprotein A (*lpnA*) gene (231 bp) was amplified by conventional PCR and hybridized with specific probes by reverse-line blotting (RLB) as previously described (Escudero et al., 2008).

For real-time PCR using tul4, ISFtu2, type A and type B assays, a type B positive control was used, as type A strains are restricted to North America.

In the four positive cultures of *F. tularensis*, the isolates were subjected to whole-genome sequencing on an Illumina MiSeq platform after DNA extraction and library preparation using the Nextera XT Library Preparation kit (Illumina, San Diego, CA, United States), according to manufacturer’s instruction.

### 2.4 SureSelect^XT HS^ custom library design for targeted sequencing of *Francisella tularensis*

RNA oligonucleotide baits (120 bp in size, a total of 54,756 baits) contiguously spanning (i.e., no tiling) the entire genome of *Francisella tularensis* were designed based on representative publicly available genomes, and plasmids, of the four *F. tularensis* subspecies (accession numbers NC_010677.1, NZ_CP009694.1, NZ_CP009683.1, NZ_CP010104.1, NZ_CP009352.1, NZ_CP010447.1, NC_006570.2, and NZ_CP009633.1). To ensure specificity, we excluded baits with considerable homology (determined by BLASTn) with the human (Human G + T) or mouse (Mouse G + T) genomes and transcriptomes or with available nucleotide sequences from the genus *Microtus* (taxonomy ID 10053), *Oryctolagus* (taxonomy ID 9984) or *Lepus* (taxonomy ID 9980). Finally, this custom library was uploaded to Agilent’s SureDesign software (settings: Tier 3; Boosting: Balanced; Probe Precedence: Reuse Existing) and synthesized by Agilent Technologies (Santa Clara, CA, United States). The set of RNA baits sequences used in the current Target Enrichment protocol is available at https://doi.org/10.5281/zenodo.8043219.

### 2.5 SureSelect^XT HS^ targeted whole-genome sequencing of *Francisella tularensis* directly from biological samples

In parallel to culture attempts, we used the designed RNA oligonucleotide baits in order to capture and sequence *F. tularensis* genomes directly from the biological samples, similarly to previous studies (Pinto et al., 2016; Borges et al., 2021; Macedo et al., 2023). After DNA extraction (described above), whole-genome capture and sequencing of *Francisella tularensis* was performed following SureSelect XT HS Target Enrichment System for Illumina Multiplexed Sequencing protocol (G9702-90000, Version E0, November 2020, Agilent Technologies, Santa Clara, CA, United States) using the custom baits library described above, in a 1:5 dilution. Library preparation started with the fragmentation of high quality DNA using the Agilent’s SureSelectXT HS Low input Enzymatic Fragmentation kit (“Method 2: Enzymatic DNA Fragmentation” option described in “Step 2.”), and was further carried out following the manufacturer’s instructions. Libraries fragments size, concentration and molarity were determined on a Fragment Analyzer system (Agilent, Santa Clara, CA, United States). Libraries were then sequenced on an Illumina MiSeq or NextSeq apparatus (Illumina, San Diego, CA, United States) (Supplementary Table 1). To evaluate the success and feasibility of the target capture and enrichment (TCE) on a routine surveillance basis (in terms of both time and costs), a single library was prepared from each sample and sequenced only once (despite the potential gains of resequencing). The sequencing reads (only reads mapping against *F. tularensis* strain FTNF002-00 genome) generated in the present study were deposited in the European Nucleotide Archive (ENA) (BioProject PRJEB63267). Detailed ENA accession numbers are described in Table 1.

**Table 1:** Details of the 17 *Francisella tularensis* genome assemblies generated in this study.

									Isolates metadata		
Sample ID	Approach	Assembly size (bp)	Assembly mean depth of coverage	No. Contigs (final assembly)	No. excluded contigs	N50	Genetic clade*	Acc. no. raw reads**	Location	Collection date	Host
FT_L09	SS^XT HS^	1,764,191	442.1	138	0	20,843	B.49†	ERR11573837	Valladolid	2016	*L. granatensis*
FT_L10	SS^XT HS^	1,766,793	415.4	128	0	22,699	B.49†	ERR11573838	Valladolid	2016	*L. granatensis*
FT_L11	SS^XT HS^	1,760,164	426.0	125	0	23,357	B.108	ERR11573839	Valladolid	2016	*L. granatensis*
FT_L36	SS^XT HS^	1,755,295	477.4	126	0	22,725	B.49	ERR11573840	Valladolid	2016	*L. granatensis*
FT_L51	SS^XT HS^	1,773,693	475.7	130	0	22,725	B.153	ERR11573841	Palencia	2017	*L. granatensis*
FT_L52	SS^XT HS^	1,775,015	577.4	125	0	22,726	B.266	ERR11573842	Palencia	2017	*L. granatensis*
FT_L71B	SS^XT HS^	1,763,718	308.1	132	0	22,699	B.108	ERR11573843	Palencia	2019	*L. granatensis*
FT_L72B	SS^XT HS^	1,774,977	357.1	153	0	20,929	B.153	ERR11573844	Palencia	2019	*L. granatensis*
FT_MA1830	SS^XT HS^	1,780,847	1316.3	114	0	26,515	B.110	ERR11573845	Palencia	2014	*M. arvalis*
FT_MA1992	SS^XT HS^	1,762,592	226.7	385	1,149	8,822	B.110	ERR11573846	Palencia	2014	*M. arvalis*
FT_MA2111	SS^XT HS^	1,725,569	319.1	242	182	12,196	B.110	ERR11573847	Palencia	2014	*M. arvalis*
FT_MA2129	SS^XT HS^	1,776,150	1117.4	114	0	25,220	B.110	ERR11573848	Palencia	2014	*M. arvalis*
FT_MA2136	SS^XT HS^	1,718,841	104.7	274	277	10,565	B.110	ERR11573849	Palencia	2014	*M. arvalis*
FT_MA2129-Lung	Culture	1,789,044	147.4	103	NA	26,622	B.110	ERR11573850	Palencia	2014	*M. arvalis*
FT_MA2129-Spleen	Culture	1,788,868	142.6	101	NA	26,986	B.110	ERR11573851	Palencia	2014	*M. arvalis*
FT_MA1953-Liver	Culture	1,788,790	158.3	101	NA	26,986	B.110	ERR11573852	Palencia	2014	*M. arvalis*
FT_MA1953-Spleen	Culture	1,788,964	108.0	102	NA	26,622	B.110	ERR11573853	Palencia	2014	*M. arvalis*

SS^XT HS^ – SureSelect ^XT HS^ target and enrichment protocol. NA, Not applicable.

*Genetic clade determined by CanSNPer2.

**Published reads of samples processed with SS^XT HS^ correspond only to raw reads mapping against the reference genome *Francisella tularensis* subsp. *holarctica* FTNF002-00 (NC_009749.1).

^†^Samples FT_L09 and FT_L10 have a mixed infection with two genotypes – clade B.49 (major population) and B.110 (minor population). See main text and Supplementary Figure 7.

### 2.6 Sequence data analysis

To assess the efficiency of the whole-genome capture and enrichment, we determined the percentage of reads that corresponded to *F. tularensis* in each sample (percentage of reads “on-target”) by mapping the reads (before and after quality improvement) against the reference genome *Francisella tularensis* subsp. *holarctica* FTNF002-00 (acc. no. NC_009749.1) using bowtie2 v.2.4.2 (Langmead and Salzberg, 2012). The percentage of the reference genome covered and respective mean depth of coverage were evaluated using the *getCoverage* tool.^1^

The *de novo* assembly of the genomes was performed with INNUca v.4.2.2,^2^ which, after reads’ quality analysis (FastQC v0.11.5)^3^ and cleaning (Trimmomatic v0.36) (Bolger et al., 2014), performs *de novo* assembly with SPAdes v3.14.0 (Bankevich et al., 2012) and post-assembly optimization with Pilon v1.23 (Walker et al., 2014). The reads and contigs were taxonomically classified using Kraken 2 v2.0.7-beta (Wood et al., 2019) with the Standard-16 16 GB database (26^th^ September 2022, available at: https://benlangmead.github.io/aws-indexes/k2). For the samples with a flag in the INNUca pipeline indicating that other taxa besides *Francisella* were identified in the assembly, only the contigs classified within the family *Francisellaceae*, or at lower taxonomical ranks, were kept in the final assemblies. When needed, genome annotation was performed with Prokka v1.14.6 (Seemann, 2014). The de novo assemblies generated in this study are available at https://doi.org/10.5281/zenodo.8043219.

### 2.7 Genetic diversity of the captured genomes and phylogenetic analysis

The genomes were genotyped using CanSNPer2,^4^ which assigns the genomes to genetic clades based on the identification of established canonical single nucleotide polymorphisms (canSNPs) (Lärkeryd et al., 2014). To assess the phylogenetic context of the newly sequenced genomes into the *F. tularensis* diversity, all the *F. tularensis* complete genomes available in RefSeq and Genbank (and associated metadata), as of 27th February 2023, were collected and also classified with CanSNPer2. The details of the genomes can be found in Supplementary Table 2, including BioProjects and associated publications (La Scola et al., 2008; Barabote et al., 2009; Antwerpen et al., 2013; Coolen et al., 2013; Alm et al., 2015; Atkins et al., 2015; Dwibedi et al., 2016; Madani et al., 2017; Busch et al., 2018; Koene et al., 2019; Sichtig et al., 2019; Busch et al., 2020; Kevin et al., 2020; Kittl et al., 2020; Myrtennäs et al., 2020; Witt et al., 2020; Neubert et al., 2021; Öhrman et al., 2021; Pisano et al., 2021).

Multi-genome alignments and extraction of single nucleotide variant sites (SNVs) was performed with Parsnp v1.2 (Treangen et al., 2014) with the parameters *–c* and *–C 2000*, using the genome of strain FTNF002-00 (acc. no. NC_009749.1) as reference (for increased phylogenetic resolution, other reference genomes were used for some subclades phylogenetic trees; see figures legends for further information). Maximum likelihood trees were obtained with MEGA-CC v10.0.5 (Kumar et al., 2018) with 100 bootstraps. For particular comparative analyses, Snippy v4.5.1^5^ was used for mapping and variant calling directly from the sequencing reads (with default parameters, with exception of *–mapqual* that was set to 20) and lofreq^6^ was run over snippy BAM files for the detection of minor variants (Wilm et al., 2012), with *indelqual* mode with *–dindel* to assess indel qualities and then *call* mode, including *–call-indels*. Only the minor variants at a frequency of ≥5%, supported by at least 4 reads in positions covered by at least 10 reads were considered and further inspected with the Integrative Genomics Viewer (IGV) for confirmation/exclusion (Thorvaldsdottir et al., 2013).

Grapetree^7^ (Zhou et al., 2018) and Microreact^8^ (Argimón et al., 2016) were used for phylogenies and metadata visualization.

## 3 Results

### 3.1 Dataset characterization and culture of samples

A total of 63 samples, including samples from 50 common voles and 13 Iberian hares, that consisted of pooled of spleen and liver homogenates, were subjected to the SureSelect^XT HS^ target capture and enrichment protocol (TCE) after DNA extraction. The counts and concentration of genome equivalents (GE) were assessed for 52 samples (46 voles and 6 hares) based on the screening of *tul4* and *ISFtu2* (Supplementary Table 1). All samples tested positive for *ISFtu2*, but 11 were negative for *tul4* in the real time PCR, but positive by conventional PCR followed by RLB. This discrepancy is likely due to the fact that ISFtu2 is an insertion element-like sequence present in multiple copies and hence its detection is more sensitive than *tul4* (Versage et al., 2003). All samples were confirmed as *F. tularensis subsp. holarctica*.

In parallel, culture was attempted on different tissues collected from the 50 voles (Supplementary Table 1). It was possible to isolate *F. tularensis* in two animals (4%), specifically from the spleen and liver of vole FT-MA1953 and from the lung and spleen of vole FT-MA2129. Concordantly, FT-MA1953 and FT-MA2129 were the voles samples with the highest GE counts used for TCE input (Supplementary Table 1). The four isolates were subjected to WGS and included in the downstream analysis.

### 3.2 Target capture and enrichment success

**Figure 1:**
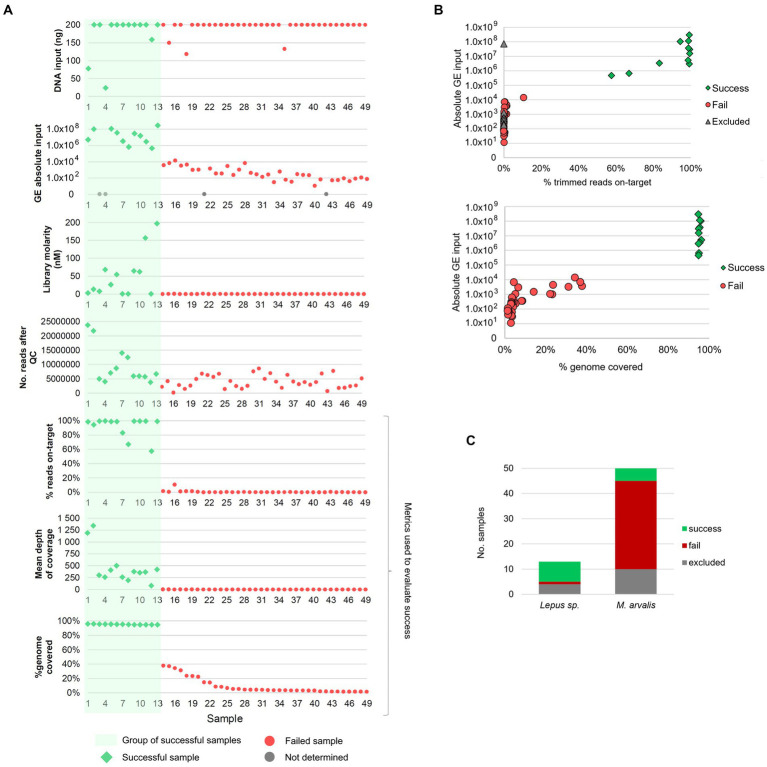
Overview of the success of the target and enrichment sequencing approach and its relation to multiple sample features. **(A)** Relation between genome coverage and percentage of reads on-target (three bottom panels), and multiple factors: initial DNA input, absolute input of genome equivalents (GE), NGS library molarity and number of reads after quality control (QC). Samples are ordered from 1 to 49 (on the *xx*-axis) from the sample with the highest to the lowest percentage of horizontal genome coverage (by at least 1-fold). **(B)** Association between absolute GE input [available for 52 samples (45 sequenced and 7 excluded)] and: (i) the percentage of reads on-target (upper panel) and, (ii) the percentage of genome coverage (lower panel). Gray triangles in the upper panel identifies the samples that were excluded before sequencing due to low library concentration but for which GE counts were available (*n* = 7). The excluded sample with high GE counts (1×10^8^) corresponds to sample FT-MA1953. **(C)** Distribution of successful, failed and excluded samples among the samples from common voles (*M. arvalis*, *n* = 50) and Iberian hares (*L. granatensis*, *n* = 13).

All 63 samples met the minimum required DNA input (10 ng) for the TCE protocol, with the majority having the maximum quantity of 200 ng of DNA available to use as input. After the TCE protocol, 14/63 samples were excluded due to very low library concentrations and the remaining 49 samples proceeded to NGS (Supplementary Table 1). A mean of 5,888,432 reads was generated per sample, with a high variability between samples (range: 169366–24,726,074 reads), although not correlating with success (Figure 1).

To evaluate the success of the TCE approach, the proportion of reads corresponding to *F. tularensis* (reads on-target) and the completeness of the genome (percentage of the reference genome covered by at least 1-fold) was determined for each sample. Among the 49 samples sequenced, 13 (26.5%) samples yielded >50% reads on-target (10 of which having >90% of reads on-target) and > 94% of the reference genome covered (Figure 1). Most of the remaining 36 unsuccessful samples had very low percentages of reads on-target (ranging between 0.01 and 10.64%, mean = 0.61%) and a mean of 9.3% of the genome covered (Figure 1). Interestingly, this revealed an “all-or-nothing” scenario where the successful and unsuccessful samples are clearly separated in two distinct groups (Figure 1B), rather than showing a linear trend of the evaluated metrics. In fact, success was obtained for all samples with more than 1 × 10^5^ GE of input, with a minimum of 463,513 GE for sample FT-MA2136, while the highest GE input among the “fail” group was 13,948 GE (Figure 1B). This large “gap” in GE input values between success and fail groups might help explain the “all-or-nothing” scenario with no samples with intermediate success in between.

Among the 50 vole samples that were also subjected to culture, isolation of *F. tularensis* was possible for two samples (4%), while the culture-free TCE approach allowed obtaining the genome of *F. tularensis* from five samples (10%). Of note, one of the two successfully cultured samples, FT-MA1953, did not proceed to NGS after the TCE protocol due to a very low library concentration. However, the high GE count (~7×10^7^) of FT-MA1953 falls within the successful GE input range and it is, in fact, the only sample failing within this group (Figure 1B). As such, we speculate that some experimental issue might have occurred during the wet-lab protocol leading to a poor library (e.g., failing to add a reagent) for this sample.

Of note, TCE success rate was much higher among hares (61.5%) than voles (10%) samples (Figure 1C), consistent with the higher GE counts observed in the former species (Supplementary Table 1).

### 3.3 Characterization of *Francisella tularensis* genomes and integration into the global phylogeny

#### 3.3.1 CanSNP classification

**Figure 2:**
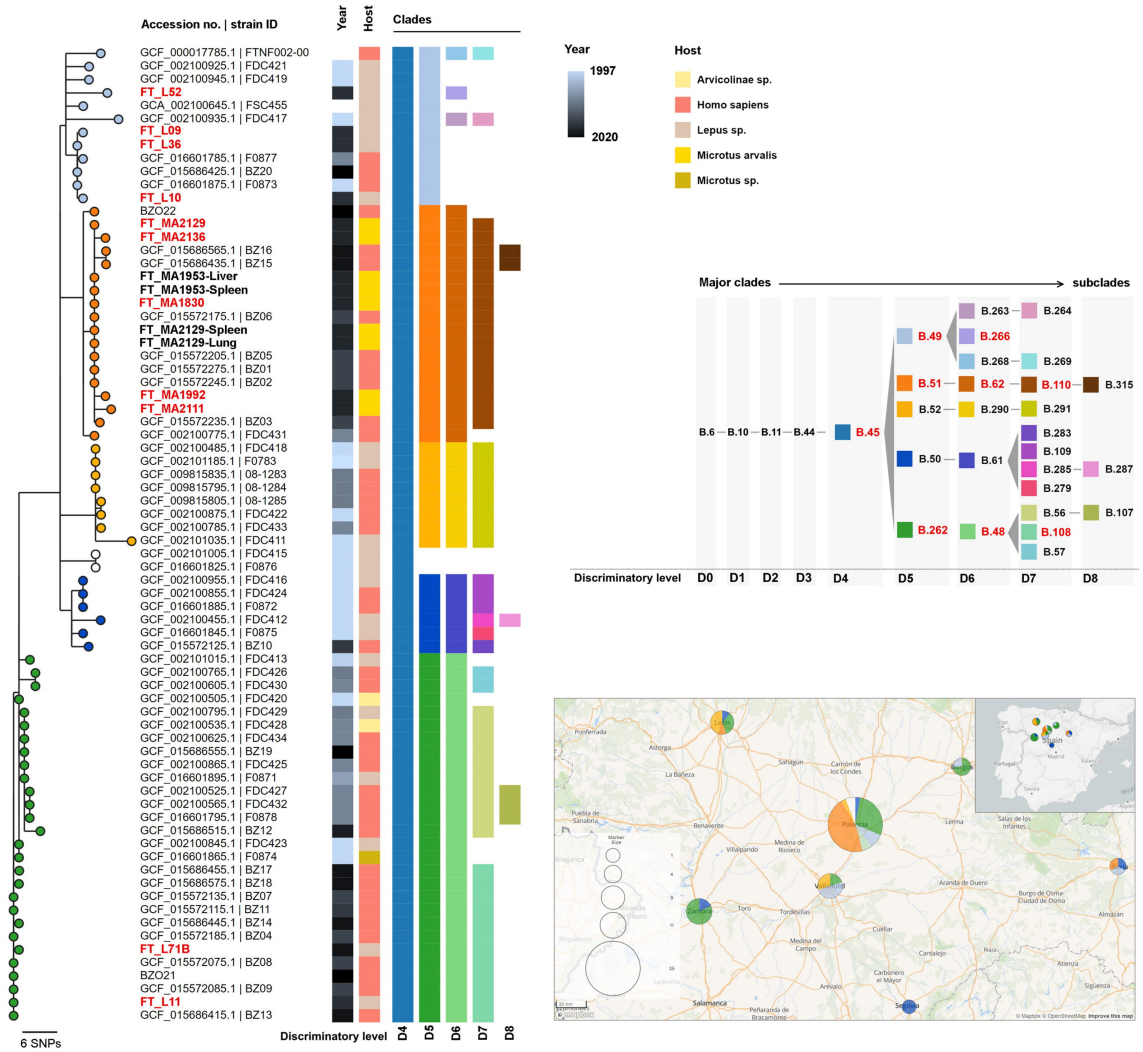
Maximum likelihood phylogenetic tree of the newly sequenced genomes from clade B.10 (D1; *n* = 17) and all available *Francisella tularensis subsp. holarctica* genome assemblies from Spain (*n* = 58; Supplementary Table 2). The phylogenetic tree was generated using MEGA-CC v10.0.5 with 100 bootstraps based on 113 core single nucleotide variant positions (SNVs) extracted from a multiple genome alignment with 1,558,560 bp, generated with parsnp using the genome of strain FTNF002-00 (acc. no. NC_009749.1) as reference. Tree nodes are colored by clade (at level D5) and metadata blocks show the respective year of collection, host and canSNP clades and subclades (from level D4 to D8). The strain IDs from samples generated in this study using the capture and enrichment approach (TCE) (*n* = 11) are shown in red. Other genomes obtained from strains isolated in culture are shown in black and bold (*n* = 4). The panel on the right shows the color codes for each clade and subclade identified among the analyzed genomes and the ancestry relationships between them. The clades identified among the TCE genomes are highlighted in red. The ancestral clades (SNP path) of major clade B.45 identified by CanSNPer2 are B.1-B.2-B.3-B.5-B.6-B.10-B.11-B.44-B.45 (not shown). The lower panel on the right shows the geographical distribution of the samples, with the same sample color scheme of the nodes on the phylogenetic tree (clade level D5). A smaller map shows the geographical location of the study area within the Iberian Peninsula.

The *de novo* assemblies of the 13 successful sequenced genomes ranged from 1.72 to 1.78 Mb, which is close to the expected *F. tularensis* genome size (1.8–1.9 Mb), and had a mean depth of coverage of 505-fold (range 104–1,316-fold) (Table 1). Genomes were typed based on canSNPs classification by canSNPer2 (Lärkeryd et al., 2014). For clarity reasons, the clades are referred to according to their level of discrimination (from D0 to D9) within this dataset (Figure 2), where level D0 corresponds to the level of the clade that is common to all the analyzed sequences and level D1 is the first level where the analyzed sequences start being discriminated by clade.

All 13 genomes belonged to major clade B.6 (level D0) (descendant of ancestral clades B.1-B.2-B.3-B.5), consistent with strains circulating in Europe and North America (Pilo, 2018). Among these, 11 genomes belonged to subclade B.10 (D1), common in Western Europe, and the other two belonged to B.7 (D1), a common clade in the United States and Scandinavia (Supplementary Figure 1) (Pilo, 2018).

#### 3.3.2 Integration in global phylogeny

All the genome assemblies of *F. tularensis* subs. *holartica* (or *F. tularensis* without subspecies described) obtained from public databases (RefSeq and Genbank) were classified with CanSNPer2. All the non-duplicate genomes from subclades B.10 and B.7 (level D1) were selected for the phylogenetic analysis (*n* = 556, Supplementary Table 2, Supplementary Figure 1).

The 11 B.10 (level D1) genomes belonged to subclade B.45 (level D4) and could be further discriminated into three subclades within level D5: (i) B.49 (*n* = 4), (ii) B.51 (*n* = 5) and (iii) B.262 (*n* = 2) (Figure 2 and Supplementary Figure 2, Table 1).

Within the global phylogeny of subclade B.49 (*n* = 81) (Supplementary Figure 3), which included mostly genomes from France (n = 45), Germany (*n* = 21) and Spain (*n* = 11), the genomes from samples FT_L09, FT_L10, and FT_L36 segregated in a branch together with other strains from Spain (three human isolates) and one from France (isolated from hare) (Figure 2 and Supplementary Figure 3). Strain FT_L52 integrates another phylogenetic branch that is composed of six B.266 (level D6) genomes, four of which are from France (including one human isolate).

Within B.51 (level D5), five genomes obtained through the TCE approach (FT_MA1830, FT_MA1992, FT_MA2111, FT_MA2129, and FT_MA2136) belonged to B.62 (level D6) and B.110 (level D7) subclades. The B.1.110 subclade (total of 19 sequences) also included the four isolates obtained from culture (FT_MA1953-Liver, FT_MA1953-Spleen, FT_MA2129-Lung, and FT_MA2129-Spleen), seven human isolates from Spain and three hare isolates from Germany (Supplementary Figure 4). Interestingly, although B.110 is strongly associated with samples collected in Spain, its ascendant clade B.51 (level D5; *n* = 59) is mostly associated with genomes from Germany (Supplementary Figure 4). Furthermore, it is noteworthy that all the B.110 strains are very closely related, even when comparing strains from Spain and Germany.

Within B.262 (level D5), both studied samples (FT_L11 and FT_L71B) belonged to subclade B.108 (D7), which involves nine other human isolates from Spain (Figure 2 and Supplementary Figure 5). This subclade is a descendent of B.48 (level D6), which is almost exclusively associated with samples collected in Spain (Supplementary Figure 5), concordant with previous reports (Dwibedi et al., 2016).

The two B.7 (level D1) genomes obtained in this study, FT_L51 and FT_L72B [further classified in subclades B.133 (D2), B.81 (D3), B.136 (D4), and B.153 (D5)], were analyzed together with 26 other genome assemblies available for this major clade (Supplementary Figure 1). These genomes originate from Norway, Germany, United States, Sweden, Finland and Russia, making FT_L51 and FT_L72B the only samples from Spain among this clade. Supplementary Figure 6 shows the phylogenetic relationship of the 22 closest isolates (USA genomes are not shown as they cluster apart at a high genetic distance).

#### 3.3.3 Comparing the genomic information obtained by TCE and culture

Among the studied sample dataset, we could obtain the genome of *F. tularensis* from both culture and TCE approaches for vole FT_MA2129. No phylogenetic differences were observed between the three genomes (FT_MA2129 obtained by TCE and the culture isolates obtained from the lung FT_MA2129-Lung and spleen FT_MA2129-Spleen) obtained from this vole (Figure 2 and Supplementary Figure 4), which could be confirmed by further fine-tuned mapping-based SNP calling. Furthermore, the TCE-derived *de novo* assembly of sample FT_MA2129 was only about ~12 kb shorter (with similar number of contigs and N50) than the assemblies generated from the cultured isolates (Table 1). Although no TCE genome was obtained from vole FT_MA1953, a mapping-based SNP analysis was also applied to compare the two culture genomes from this animal (FT_MA1953-Liver and FT_MA1953-Spleen), confirming that they had no differences.

To further access the genomic information obtained with TCE, we explored the minor variants (i.e., low frequency populations) in sample FT_MA2129. For this, we mapped the reads of all three samples FT_MA2129, FT_MA2129-Spleen, and FT_MA2129-Lung against the FT_MA2129-Lung genome assembly and detected the minor variants with ≥5% frequency. We validated 13 minor variants in sample FT_MA2129 (with frequencies ranging between 5.1% and 13.8%) that were not detected in either of the two culture-derived genomes (which had only one minor variant validated each) (Supplementary Table 3). The proteins predicted to be affected by the 13 mutations include several transporters and two proteins potentially involved in host-pathogen interaction (ankyrin repeat domain-containing protein and normocyte binding protein 2b), among others (Supplementary Table 3).

#### 3.3.4 Detection of mixed infections

The screening of minor variants was also performed for the remaining samples and revealed multiple mutations at frequencies between 20 and 50% in samples FT_L09 and FT_L10. To assess if these mutations corresponded to subpopulations of the same strain or to a different strain in the same sample, we artificially inserted the minor mutations in the respective original assemblies. The CanSNPer2 classification of these assemblies revealed that the minor populations belonged to clades B.110 (D7) (descendant of B.51 (D5) and B.62 (D6)) contrarily to the major populations in these samples that belong to genotype B.49 (D5), confirming that the two hares had mixed infections with two different genotypes. Moreover, the integration of these genomes (FT_L09_MINOR and FT_L10_MINOR) in the phylogenetic analysis of the dataset from Spain show that they cluster perfectly within clade B.110 (Supplementary Figure 7), together with the other five TCE genomes already in this clade.

## 4 Discussion

In this study, we were able to sequence near-complete *F. tularensis* genomes using a culture-free approach relying on the use of RNA oligonucleotide “baits” to capture and enrich *F. tularensis* genomic material directly from complex biological samples. The high-quality sequencing data generated allowed the characterization and typing of the newly sequenced genomes and their integration into the global phylogeny of *F. tularensis subsp. holarctica*. The new genomes belonged to clades B.10 (D1), common in Western Europe, and unexpectedly to B.7 (D1), common in the United States and Scandinavia (Pilo, 2018).

The TCE approach here described had a higher success rate than the conventional culture-based method. A recent study by Wagner et al. (2022) has applied a similar approach with Agilent SureSelect technology (Agilent Technologies, Santa Clara, CA, United States) where RNA probes were designed to detect and characterize the genome of *F. tularensis* and other diverse species in the family *Francisellaceae*. Although a high success among samples with low concentration of target DNA (including environmental samples) was reported, there was no information regarding a direct or indirect quantification of the pathogen in the samples, which hampers a direct comparison with the success rate achieved in the present work. Furthermore, Wagner and colleagues processed the samples with two rounds of TCE, considerably increasing processing time and cost (Wagner et al., 2022). Still, as a potential gain in sensitivity may be achieved, the TCE two-round strategy applied by Wagner and colleagues may be a very interesting option. Our approach had an estimated cost of ~250€ per sample (for SureSelect and Illumina reagents only) and a turnaround time of four to five working days (from sample processing to sequence data), presenting a valuable option to recover near-complete genomic information.

The implementation of a TCE protocol also largely benefits from a pre-selection of samples with higher probability of success. Here, we showed that samples with at least 1 × 10^5^ GE of input had a success rate close to 100%. As such, the GE concentration estimated from real-time PCR data provides a straightforward approach for sample pre-selection using this “cut-off” value, increasing the effectiveness of the protocol.

Of note, we observed a higher success rate of TCE sequencing of hares samples (which had much higher GE concentrations) compared to voles samples. One explanation for this might be the capture context of these animals since, unlike voles (that were captured with traps), some of the sampled hares might have died of tularemia (7/13 were found dead) and others were hunted or hit by car, hinting that they might have been debilitated. Thus, in a pure speculative basis, if these animals died (or were sick) with tularemia, it is likely that they had much higher bacterial loads (congruent with our observations).

Comparing the sequence data generated from the same sample with both TCE and culture-based methods (sample FT_MA2129) showed that the designed RNA baits are highly efficient to capture the full genomic information of *F. tularensis* and that the culture-free generated data is highly reliable and equivalent to that obtained by sequencing of the isolates. This observation indicates that the 13 newly sequenced genomes could be well characterized at phylogenetic level. Importantly, CanSNP-based typing showed that the new genomes belonged to different clades commonly identified in Europe and was highly congruent with the genome-based phylogenies. Further phylogenetic analysis indicated that most of the new genomes were closely related to other genomes from Spain (Figure 2), with the noteworthy exception of the two new genomes from clade B.7, which is more commonly found in Scandinavia and the United States (Supplementary Figure 6). As previously reported, and in accordance with the known low genetic diversity of the subspecies, subclades do not generally correlate with geography or host (Dwibedi et al., 2016; Kevin et al., 2020), as is well illustrated here by the identification of several closely related strains largely dispersed in different countries and different hosts.

Interestingly, the three main clades (at the D5 discriminatory level) found in Spain, B.49, B.51, and B.262 (Supplementary Figure 2), were identified in both human and hares (B.49 and B.52; Figure 2) during the initial outbreaks (1997–2007) and later identified in humans and common voles (B.51; Figure 2) during the most recent vole outbreaks (2014 onwards). As such, our findings are consistent with the important roles of hares and voles outbreaks in the tularemia epidemiology and transmission to humans in this region (e.g., 2014 outbreak; see Ariza-Miguel et al., 2014; Luque-Larena et al., 2015; Herrero-Cófreces et al., 2021). Remarkably, using TCE sequencing, in two hares we were able to identify mixed infections with two distinct genotypes (clades B.49 and B.51), which further highlights the role of this host species in the transmission dynamics of tularemia.

Furthermore, we could also identify minor variants in a sample subjected to TCE that were not present in the genomes obtained from the respective cultured isolates. We hypothesize that these subpopulations reflect the within-host pathogen diversity, potentially linked to the ongoing adaptation to different niches/tissues. While RNA “baits” acted upon complex biological samples (in this case, a homogenate of liver and spleen), potentially capturing the *in vivo* genomic diversity of the strain, sequencing from culture reflects only the diversity of one or a few colonies. This approach has been previously applied for other pathogens, such as *Treponema pallidum*, revealing substantial within-patient genetic diversity (Pinto et al., 2016), *Mycobacterium tuberculosis*, as an alternative approach for surveillance and drug susceptibility inferences (Macedo et al., 2023), and *Chlamydia trachomatis*, where the technique was used to characterize and monitor the transcontinental dissemination of an emergent recombinant strain (Borges et al., 2021). In this context, despite *F. tularensis* being a highly monomorphic pathogen, the TCE approach allows the exploration of this additional layer of genetic variability of low-frequency populations and potentially distinguish isolates that are otherwise indistinguishable (if only the consensus sequences are considered).

Globally, this study showed that the TCE method can generate complete *F. tularensis* genomic information in a timely manner, allowing us to carry out highly discriminatory phylogenetic analysis at whole-genome level. We performed CanSNP typing, integration of genomes into global phylogeny (with important observations in regards to clades geographical distribution and hosts) and fine-tuned mutation analysis, showing that TCE can greatly increase the current knowledge on *F. tularensis*. Although the applicability of target capture and enrichment for sequencing on a routine surveillance basis is still debatable (complexity of protocols, cost), TCE performed with greater success than culture-dependent sequencing and can be further potentiated by the pre-selection of samples based on real-time PCR data, as we propose. Considering the low success rate of *F. tularensis* culture and the fact that serology, a main diagnosis method, provides no information regarding molecular epidemiology, the present methodology provides a highly valuable approach toward an increased knowledge on the genomics and epidemiology of this highly infectious pathogen.

## Data availability statement

Sequencing reads (only reads mapping against F. tularensis strain FTNF002-00 genome) generated in the present study were deposited in the European Nucleotide Archive (ENA) (BioProject PRJEB63267). Detailed ENA accession numbers are described in Table 1. The set of RNA baits sequences used in the current Target Enrichment protocol and the de novo assemblies are available at https://doi.org/10.5281/zenodo.8043219.

## Ethics statement

The animal study was approved by the Ethics Committee from the University of Valladolid (authorization code: 4801646). The study was conducted in accordance with the local legislation and institutional requirements.

## Author contributions

JI: Investigation, Methodology, Writing – original draft, Data curation, Formal analysis, Validation. RE: Investigation, Methodology, Writing – original draft, Conceptualization, Funding acquisition, Project administration, Resources, Supervision. JL-L: Conceptualization, Funding acquisition, Investigation, Methodology, Resources, Supervision, Writing – review & editing. MP: Investigation, Methodology, Writing – review & editing, Data curation. VB: Data curation, Investigation, Methodology, Writing – review & editing, Validation. RG-M-N: Investigation, Writing – review & editing, Resources. SD: Writing – review & editing, Data curation, Methodology. LV: Data curation, Methodology, Writing – review & editing. FM: Methodology, Writing – review & editing, Conceptualization, Funding acquisition, Investigation, Resources, Supervision. DV: Conceptualization, Investigation, Methodology, Resources, Supervision, Writing – review & editing. DH-R: Investigation, Resources, Writing – review & editing, Data curation, Visualization. RR-P: Data curation, Investigation, Resources, Writing – review & editing. SH-C: Data curation, Investigation, Resources, Writing – review & editing. FJ-T: Resources, Writing – review & editing. JG: Conceptualization, Funding acquisition, Investigation, Methodology, Project administration, Resources, Supervision, Writing – original draft. IL: Conceptualization, Funding acquisition, Investigation, Methodology, Project administration, Resources, Supervision, Writing – original draft.

## Footnotes

^1^https://github.com/monsanto-pinheiro/getCoverage

^2^https://github.com/B-UMMI/INNUca

^3^http://www.bioinformatics.babraham.ac.uk/projects/fastqc/

^4^https://github.com/FOI-Bioinformatics/CanSNPer2

^5^https://github.com/tseemann/snippy

^6^https://github.com/CSB5/lofreq

^7^https://github.com/achtman-lab/GrapeTree

^8^https://microreact.org/
