# Supplementary material for: Strengthening the genomic surveillance of Francisella tularensis by using culture-free whole-genome sequencing from biological samples
Source: Front Microbiol. 2024 Jan 5;14:1277468. doi: 10.3389/fmicb.2023.1277468 (PMC10797068; doi:10.3389/fmicb.2023.1277468)
Supplement: Supplementary file 4 [file Data_Sheet_1.DOCX]

Supplementary Material

# Supplementary Figures


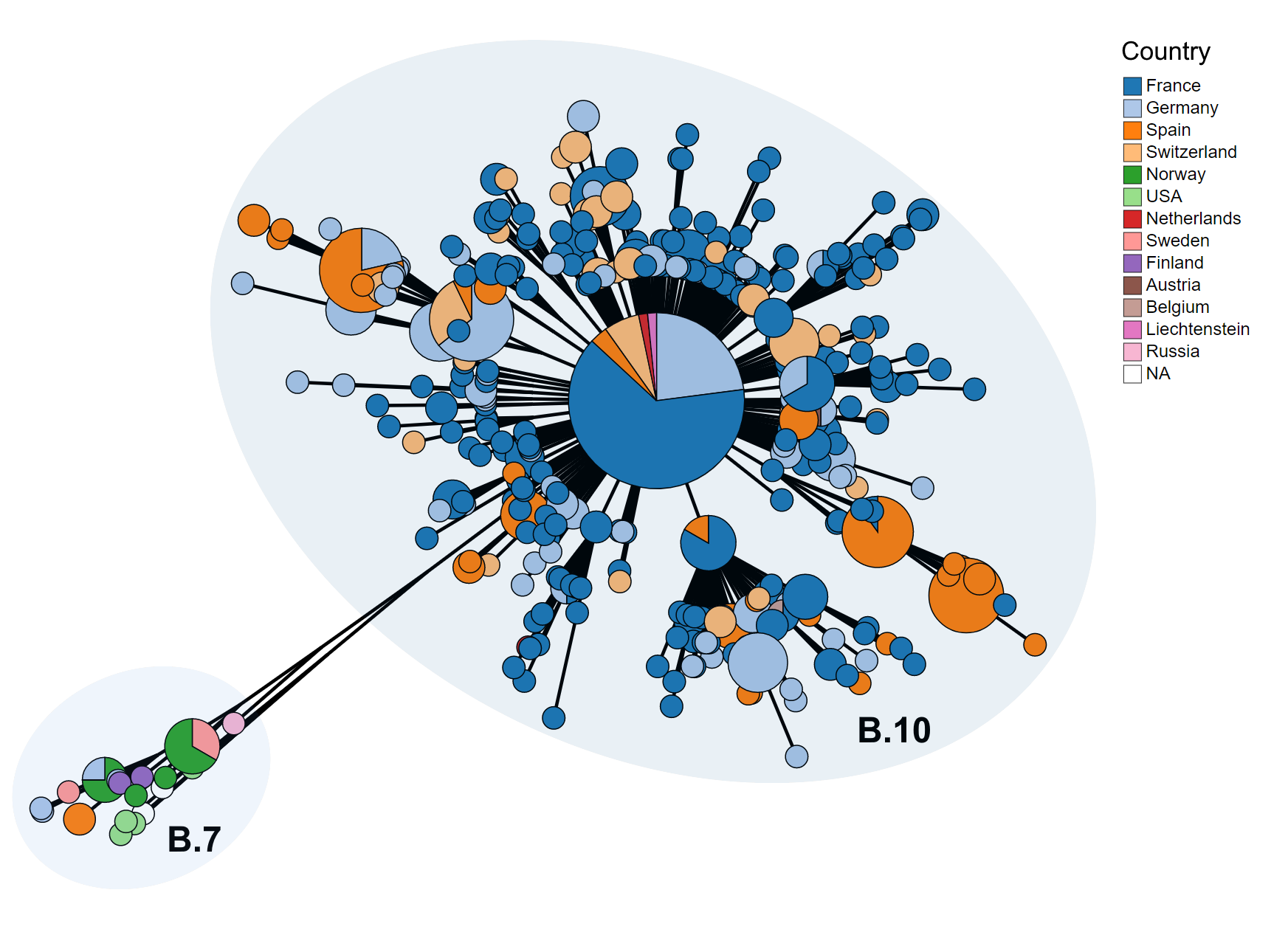


**Supplementary Figure 1**

Minimum spanning tree of the total genome dataset used in this study (n=599), coloured by major clade (level D1) (upper panel) and country (lower panel). The phylogenetic tree was generated using parsnp, based on 1137 core single nucleotide variant positions (SNVs) extracted from a multiple genome alignment with 833706 bp and using the genome of strain FTNF002-00 (acc. no. NC_009749.1) as reference.

**
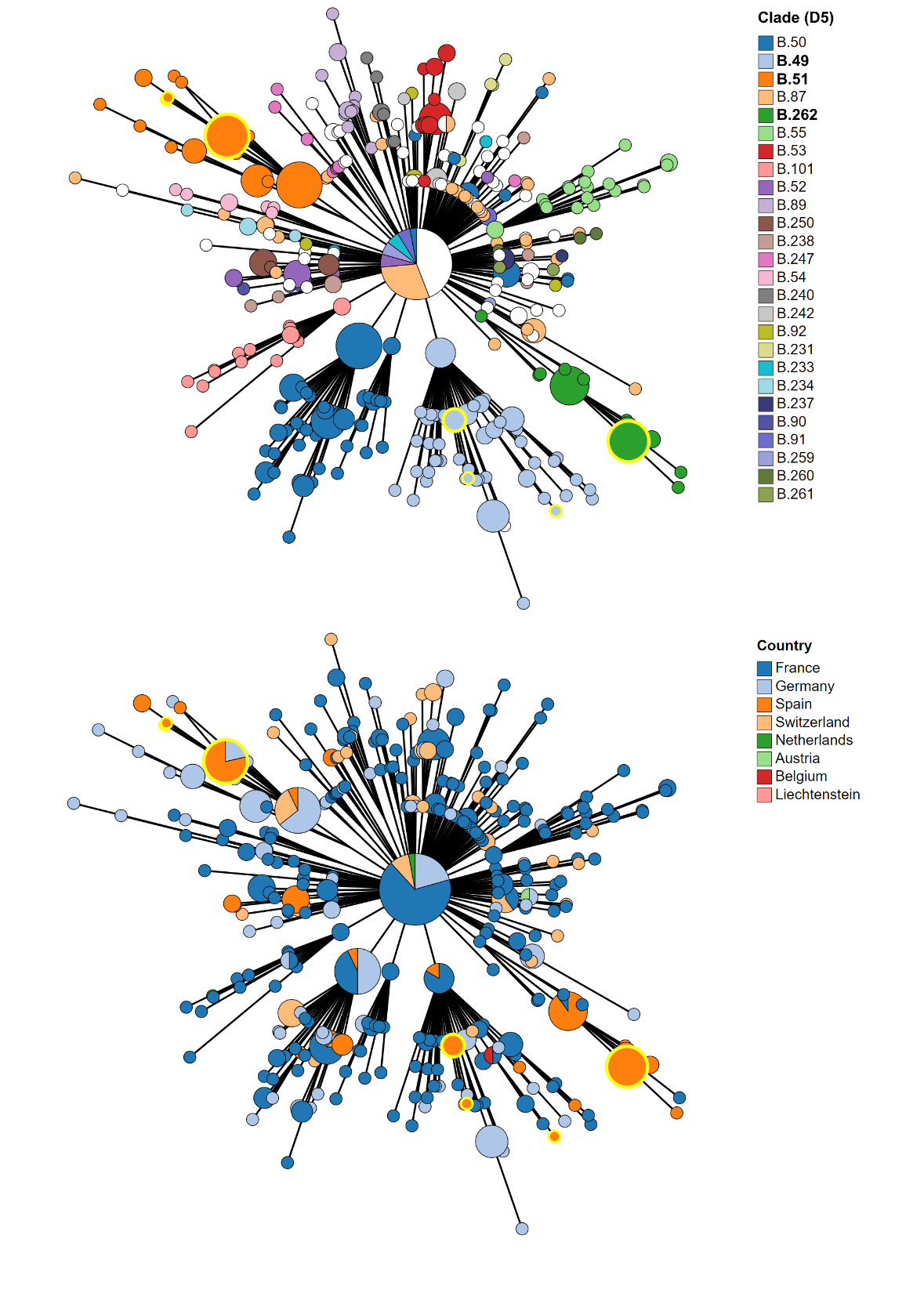
**

**Supplementary Figure 2**

Minimum spanning tree of the genomes from clade B.10 (level D.1) used in this study (n=571), coloured by subclade (D5) (upper panel) and country (lower panel). A maximum likelihood phylogenetic tree was generated using MEGA-CC v10.0.5 with 100 bootstraps based on 1124 core single nucleotide variant positions (SNVs) extracted from a multiple genome alignment with 902376 bp generated with parsnp using the genome of strain FTNF002-00 (acc. no. NC_009749.1) as reference.

**
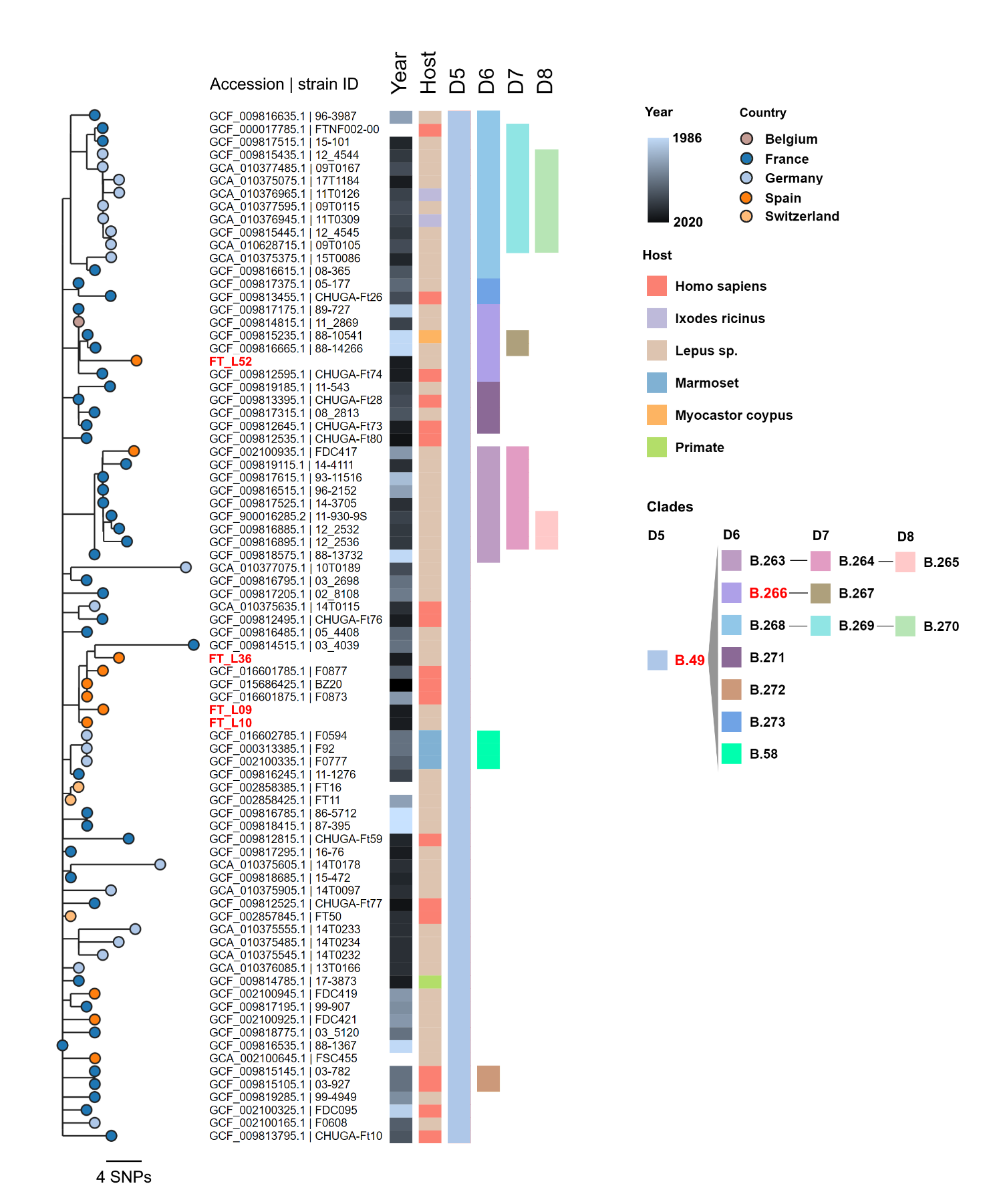
**

**Supplementary Figure 3**

Maximum likelihood phylogenetic tree of the 80 genomes from clade B.49 (level D5) analyzed in this study. The maximum likelihood phylogenetic tree was generated using MEGA-CC v10.0.5 with 100 bootstraps based on 1124 core single nucleotide variant positions (SNVs) extracted from a multiple genome alignment with 902376 bp generated with parsnp using the genome of strain FTNF002-00 (acc. no. NC_009749.1) as reference. Tree nodes are colored by country and metadata blocks show the respective year of collection, host and canSNP clades and subclades (from level D5 to D8). The strain IDs from samples generated in this study using the capture and enrichment approach are shown in red. The clades panel on the right shows the colour codes for each clade and subclade identified among the analyzed genomes and the ancestry relationships between them. The ancestral clades (SNP path) of clade B.49 identified by CanSNPer2 are B.1-B.2-B.3-B.5-B.6-B.10-B.11-B.44-B.45 (not shown).

**
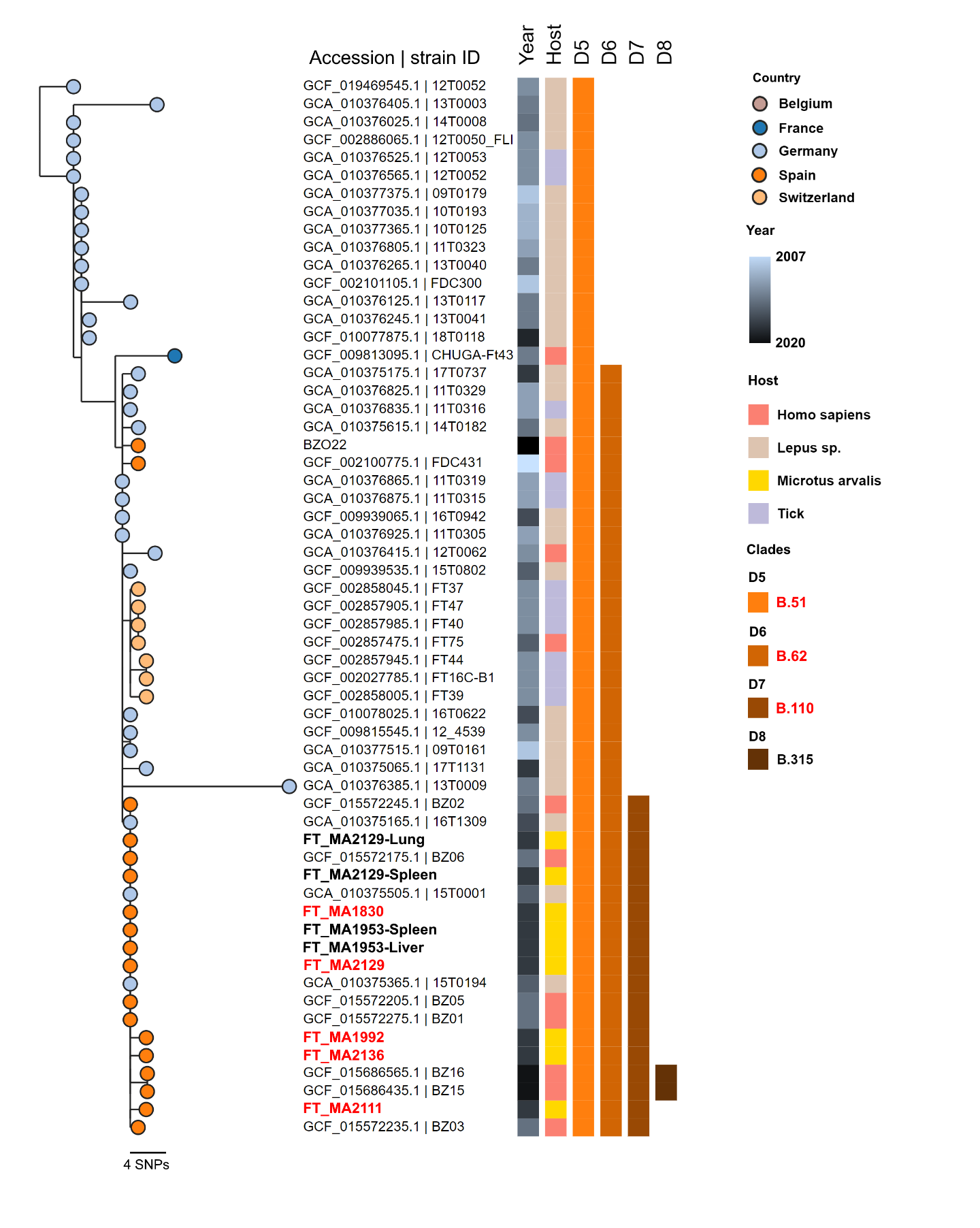
**

**Supplementary Figure 4**

Maximum likelihood phylogenetic tree of the 59 genomes from clade B.51 (level D5) analyzed in this study. A maximum likelihood phylogenetic tree was generated using MEGA-CC v10.0.5 with 100 bootstraps based on 94 core single nucleotide variant positions (SNVs) extracted from a multiple genome alignment with 1243084 bp generated with parsnp using the genome of strain 12T0052 (acc. no. NZ_CP058275.1) as reference. Tree nodes are colored by country and metadata blocks show the respective year of collection, host and canSNP clades and subclades (from level D5 to D8). The strain IDs from samples generated in this study using the capture and enrichment approach are shown in red. Other genomes obtained from strains isolated in culture are shown in black and bold. The ancestral clades (SNP path) of clade B.51 identified by CanSNPer2 are B.1-B.2-B.3-B.5-B.6-B.10-B.11-B.44-B.45 (not shown).

**
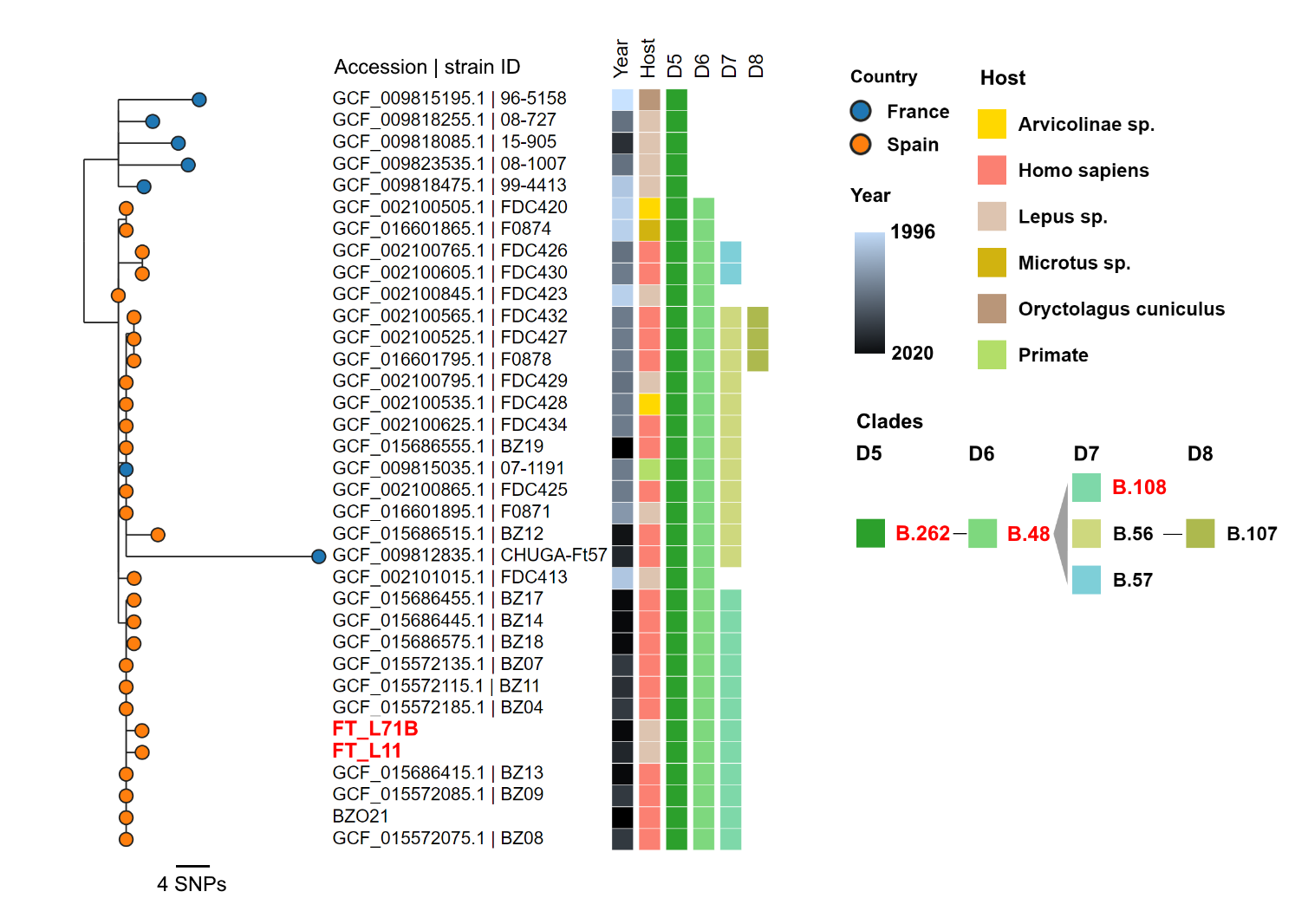
**

**Supplementary Figure 5**

Maximum likelihood phylogenetic tree of the 35 genomes from clade B.262 (level D5) analyzed in this study. A maximum likelihood phylogenetic tree was generated using MEGA-CC v10.0.5 with 100 bootstraps based on 79 core single nucleotide variant positions (SNVs) extracted from a multiple genome alignment with 1667155 bp generated with parsnp using the genome of strain FDC432 (acc. no. GCF_002100565.1) as reference. Tree nodes are colored by country and metadata blocks show the respective year of collection, host and canSNP clades and subclades (from level D5 to D8). The strain IDs from samples generated in this study using the capture and enrichment approach are shown in red. The clades panel on the right shows the colour codes for each clade and subclade identified among the analyzed genomes and the ancestry relationships between them. The ancestral clades (SNP path) of clade B.262 identified by CanSNPer2 are B.1-B.2-B.3-B.5-B.6-B.10-B.11-B.44-B.45 (not shown).

**
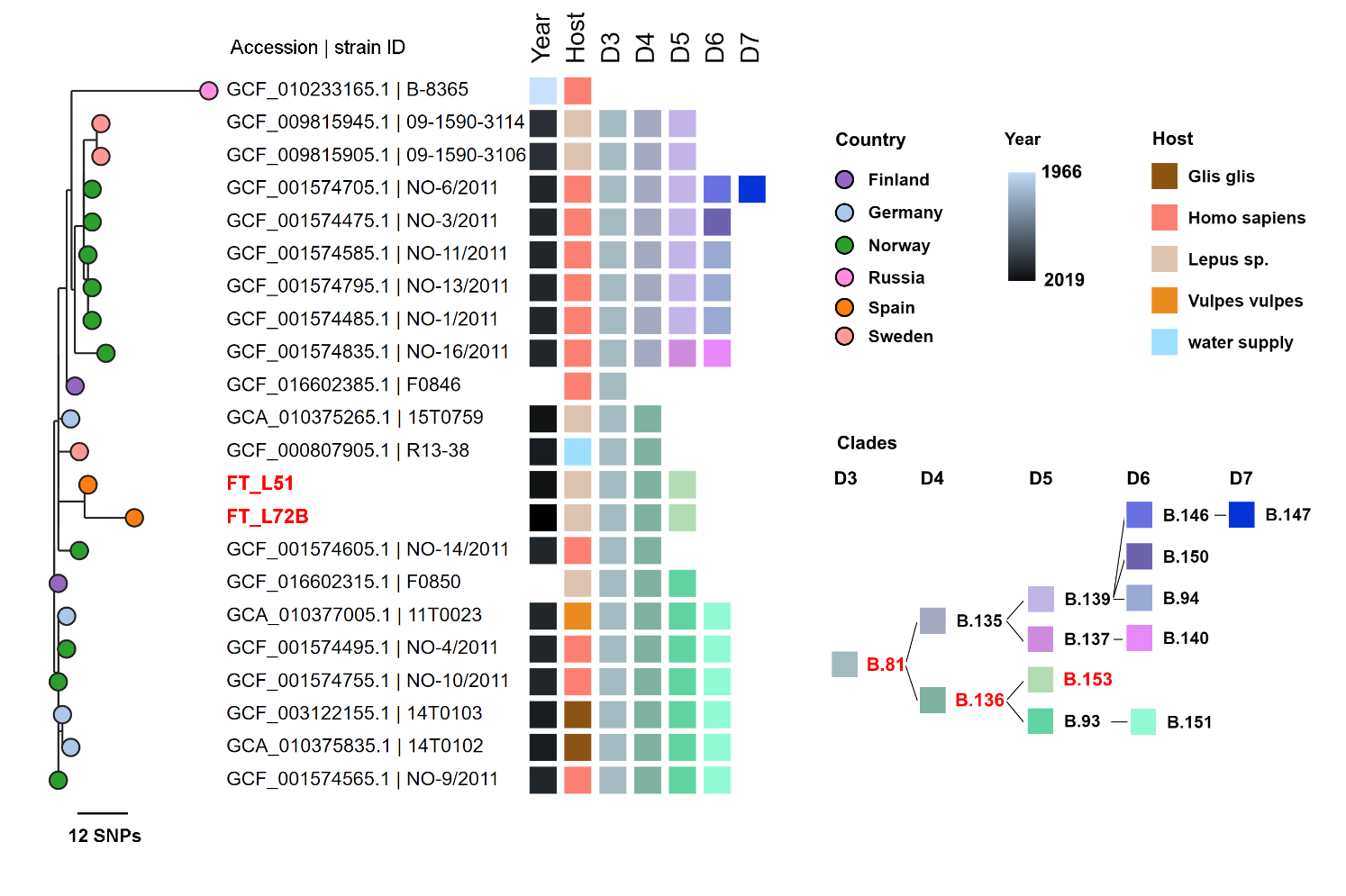
**

**Supplementary Figure 6**

Maximum likelihood phylogenetic tree of the genomes from clade B.7 (level D1) analyzed in this study. A maximum likelihood phylogenetic tree was generated using MEGA-CC v10.0.5 with 100 bootstraps based on 94 core single nucleotide variant positions (SNVs) extracted from a multiple genome alignment with 1271366 bp generated with parsnp using the genome of strain B-8365 (acc. no. NZ_CP044004.1) as reference. Tree nodes are colored by country and metadata blocks show the respective year of collection, host and canSNP clades and subclades (from level D3 to D7). The strain IDs from samples generated in this study using the capture and enrichment approach are shown in red. The clades panel on the right shows the colour codes for each clade and subclade identified among the analysed genomes and the ancestry relationships between them. The ancestral clades (SNP path) of clade B.81 identified by CanSNPer2 are B.1-B.2-B.3-B.5-B.6-B.7-B.133 (not shown).

**
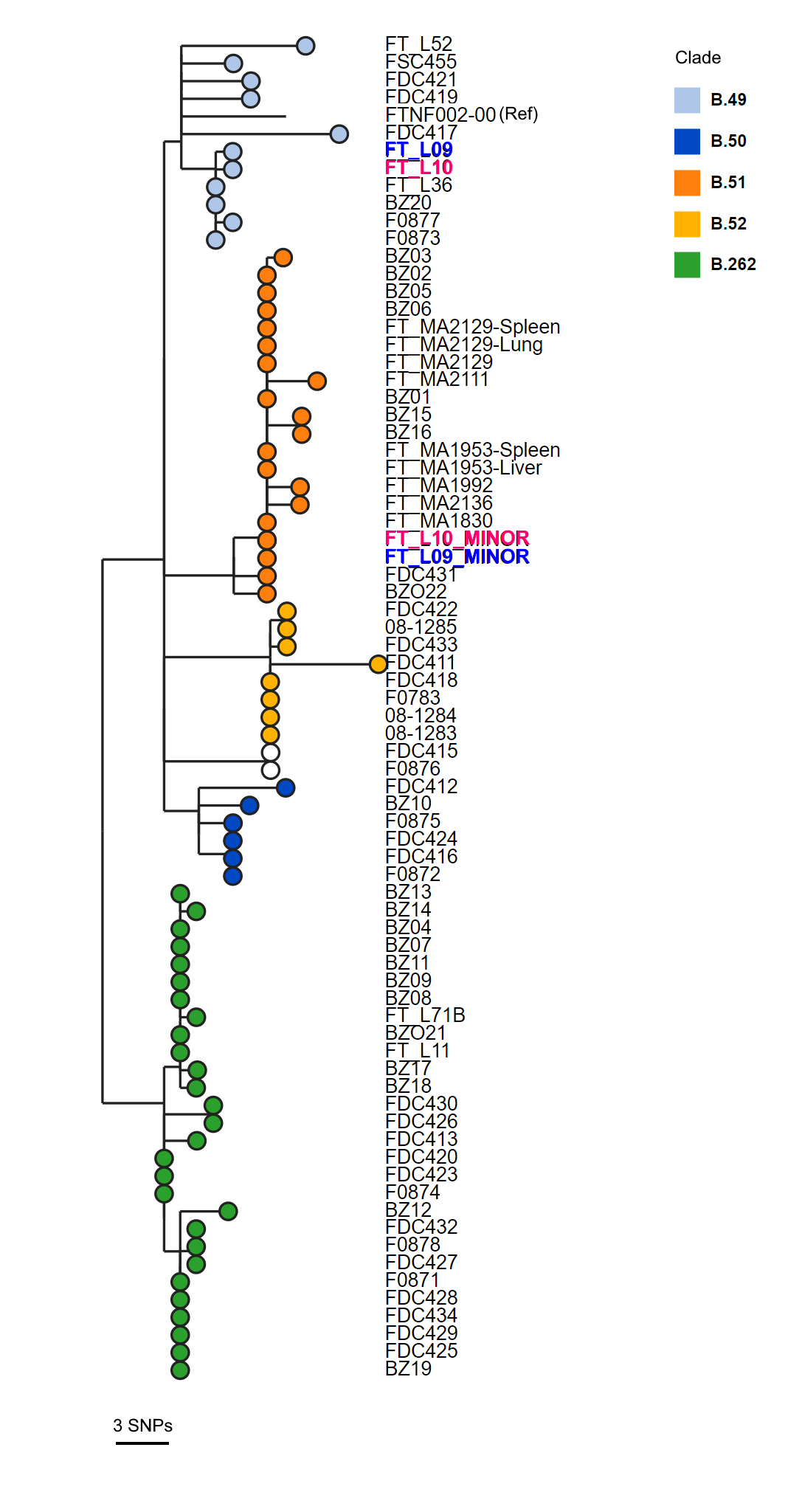
**

**Supplementary Figure 7**

Maximum likelihood phylogenetic tree of the newly sequenced genomes from clade B.10 (D1; n=17), the two assemblies corresponding to the minor populations in samples FT_L09 and FT_L10 (FT_L09_MINOR and FT_L10_MINOR) and all available *Francisella tularensis subsp. holarctica* genome assemblies from Spain (n=58; Table S2). The phylogenetic tree was generated using MEGA-CC v10.0.5 with 100 bootstraps based on 113 core single nucleotide variant positions (SNVs) extracted from a multiple genome alignment with 1558579 bp, generated with parsnp using the genome of strain FTNF002-00 (acc. no. NC_009749.1) as reference. Tree nodes are colored by clade (at discriminatory level D5).
